# Evaluating Mortality Predictors in COVID-19 Intensive Care Unit Patients: Insights into Age, Procalcitonin, Neutrophil-to-Lymphocyte Ratio, Platelet-to-Lymphocyte Ratio, and Ferritin Lactate Index

**DOI:** 10.3390/diagnostics14070684

**Published:** 2024-03-24

**Authors:** Fatma Meral Ince, Ozge Alkan Bilik, Hasan Ince

**Affiliations:** 1 Infectious Diseases and Clinical Microbiology, Selahaddin Eyyubi State Hospital, 21100 Diyarbakir, Turkey; 2 Medical Microbiology, Selahaddin Eyyubi State Hospital, 21100 Diyarbakir, Turkey; 3 Internal Medicine, Selahaddin Eyyubi State Hospital, 21100 Diyarbakir, Turkey; drhasanince@gmail.com

**Keywords:** COVID-19, mortality, neutrophil/lymphocyte ratio, procalcitonin, ferritin, lactate

## Abstract

Introduction: Numerous studies suggest that alterations in blood parameters, such as changes in platelet, lymphocyte, hemoglobin, eosinophil, and basophil counts; increased neutrophil counts; and elevated neutrophil/lymphocyte and platelet/lymphocyte ratios, signal COVID-19 infection and predict worse outcomes. Leveraging these insights, our study seeks to create a predictive mortality model by assessing age and crucial laboratory markers. Materials and Methods: Patients were categorized into two groups based on their hospital outcomes: 130 survivors who recovered from their Intensive Care Unit (ICU) stay (Group 1) and 74 who died (Group 2). We then developed a predictive mortality model using patients’ age, neutrophil-to-lymphocyte ratio (NLR), platelet-to-lymphocyte ratio (PLR), procalcitonin levels, and ferritin lactate (FL) index results. Results: A total of 204 patients were included. Patients in Group 2 had a notably higher mean age compared to those in Group 1 (76 ± 11 vs. 66 ± 15 years) (*p* < 0.001). Using specific cut-off values, our analysis revealed varying effectiveness in predicting COVID-19 mortality: Those aged over 73 years showed 74% sensitivity and 60% specificity, with an area under the curve (AUC) of 0.701. Procalcitonin levels above 0.35 ng/mL balanced true-positive and -negative identifications well, achieving an AUC of 0.752. The FL index, with a threshold of 1228 mg/dL, had 68% sensitivity and 65% specificity with an AUC of 0.707. A PLR higher than 212 resulted in 48% sensitivity and 69% specificity, with an AUC of 0.582. An NLR higher than 5.8 resulted in 55% sensitivity and 63% specificity, with an AUC of 0.640, showcasing diverse predictive accuracies across parameters. The statistical analysis evaluated the effects of age (>73), procalcitonin levels (>0.35), FL > 1228, PLR > 212, and NLR > 5.8 on mortality variables using logistic regression. Ages over 73 significantly increased event odds by 2.1 times (*p* = 0.05), procalcitonin levels above 0.35 nearly quintupled the odds (OR = 5.6, *p* < 0.001), high FL index levels more than tripled the odds (OR = 3.5, *p* = 0.003), a PLR > 212 significantly increased event odds by 3.5 (*p* = 0.030), and an NLR > 5.8 significantly increased event odds by 1.6 (*p* = 0.043). Conclusions: Our study highlights significant predictors of mortality in COVID-19 ICU patients, including advanced age, elevated procalcitonin, FL index levels, the PLR, and the NLR.

## 1. Introduction

The emergence of the Coronavirus Disease 2019 (COVID-19), caused by the SARS-CoV-2 virus, has precipitated a global health crisis of unprecedented scale and severity [1,2]. Classified as a pandemic by the World Health Organization (WHO), COVID-19 has manifested a profound threat to public health systems, economies, and societies worldwide [1,2,3]. With its rapid transmissibility and significant morbidity and mortality rates, the disease has propelled global health authorities and researchers into a relentless pursuit of understanding its pathogenesis and clinical manifestations, as well as effective management strategies [1].

The pathogenesis of COVID-19, while extensively studied, remains incompletely understood. It is known that the virus primarily targets the respiratory system, but its effects can be systemic, affecting multiple organ systems [4,5]. The virus enters host cells via the angiotensin-converting enzyme 2 (ACE2) receptor, leading to a range of clinical manifestations, from asymptomatic infection to severe respiratory and systemic complications [6]. Notably, the immune response to SARS-CoV-2 infection has been identified as a double-edged sword; while essential for viral clearance, an excessive or dysregulated immune response, termed a “cytokine storm”, can lead to tissue damage, organ failure, and, in severe cases, death [7]. This complex interplay between the virus and the host immune system underscores the challenges in managing COVID-19, particularly in patients who develop severe disease [8].

The clinical spectrum of COVID-19 is broad, encompassing asymptomatic carriers, mild upper respiratory tract infections, and severe pneumonia with acute respiratory distress syndrome (ARDS) necessitating Intensive Care Unit (ICU) admission [9,10,11,12,13,14]. The variability in disease expression is a hallmark of COVID-19, with several factors influencing disease severity and outcomes. Mortality rates among ICU-admitted patients have varied widely across different studies and populations and are reported to be between 17% and 88% [12,15,16,17,18,19]. This variability reflects not only the heterogeneity of patient populations and healthcare systems but also the dynamic nature of the pandemic and the evolving understanding of best clinical practices [11,20].

A critical aspect of managing the COVID-19 pandemic has been identifying factors predictive of severe disease, ICU admission, and mortality [15,21]. The significance of this lies not only in the potential for early intervention but also in the ability to choose the optimal treatment environment. Accurately identifying patients who are unlikely to worsen is essential for the consideration of transitioning their care to an ambulatory setting, thereby reducing the burden on the healthcare system and enhancing patient experiences. Such a strategy has been validated as both safe and highly effective in a systematic review, contingent upon the careful optimization of patient selection criteria [22]. Research has consistently highlighted several risk factors for severe COVID-19, including older age, the presence of pre-existing comorbidities (such as cardiovascular disease, diabetes, chronic respiratory disease, and cancer), obesity, and specific laboratory markers indicative of systemic inflammation or organ dysfunction [21,23,24]. Recognizing these predictors is crucial for early intervention, risk stratification, and optimizing resource allocation in overwhelmed healthcare settings.

The global research community has embarked on an extensive exploration of COVID-19, aiming to unravel its complexities and identify effective interventions [12,15]. Studies have spanned from epidemiological investigations to clinical trials, laboratory research, and public health studies, each contributing to a growing body of knowledge that informs the global response to the pandemic. Among the key areas of focus is the development of therapeutic strategies that can mitigate the severe impacts of the disease. This includes antiviral medications, immune modulators, supportive care interventions, and, critically, the development and deployment of effective vaccines. The rapid development of multiple vaccines against SARS-CoV-2 represents a monumental achievement in the fight against COVID-19, offering hope for controlling the pandemic [5,13,24].

However, the journey is far from over. The emergence of viral variants with mutations in key genomic regions has posed new challenges, potentially affecting the efficacy of existing vaccines and therapeutic agents [3,13]. Moreover, the global distribution of vaccines has highlighted disparities in access and equity, underscoring the need for international collaboration and solidarity in addressing the pandemic. The long-term impacts of COVID-19, including the physical and psychological sequelae in survivors (often referred to as “long COVID”) and the broader socio-economic disruptions, will likely be felt for years to come [3,17,19].

Several studies have demonstrated that changes in certain hematological blood parameters, including a decrease in platelet, lymphocyte, hemoglobin, eosinophil, and basophil counts; an increase in neutrophil count; and elevated neutrophil/lymphocyte and platelet/lymphocyte ratios, are indicative of COVID-19 infection and are associated with poorer clinical outcomes [10,11,12,13,14,25]. Building upon these findings, our study aims to develop a predictive model for mortality by investigating key laboratory parameters: age, procalcitonin, the NLR, the PLR, and the ferritin lactate (FL) index.

## 2. Materials and Methods

### 2.1. Patients and Design

This research received ethical approval from the Local Ethics Committee, securing approval with the number 866 on 9 September 2021. Our study adopted a retrospective analysis approach, reviewing the medical records of 204 patients who were treated in our specialized COVID-19 Intensive Care Unit (ICU) during the period from 1 June 2020 to 30 November 2020.

A confirmed diagnosis of COVID-19 was established based on the positive outcomes of real-time reverse transcriptase polymerase chain reaction (RT-PCR) assays performed on nasal and pharyngeal swab samples.

The criteria for inclusion in this study were meticulously defined to ensure a homogenous patient cohort, encompassing adults aged 18 years and above who were admitted to the Intensive Care Unit for COVID-19 treatment. All our patients were without vaccination.

The exclusion criteria were set to omit individuals with negative RT-PCR results (confirmed by two consecutive tests conducted 48 hours apart), patients who were transferred to other medical facilities, and healthcare workers who contracted the SARS-CoV-2 infection as a result of hospital exposure. All our patients had pulmonary COVID-19 only. Those with extrapulmonary COVID-19 were excluded from the study. Patients presenting with significant comorbidities, such as bacterial pneumonia, myocardial infarction (MI), cerebrovascular accidents (CVAs), or other concurrent infections, were also excluded.

For the purposes of our analysis, the patients were divided into two distinct groups based on the outcome after their hospitalization. The first group comprised 130 patients (Group 1 = Survivors) who overcame the infection and survived their ICU stay. The second group consisted of 74 patients (Group 2 = Deceased) who succumbed to the disease during their time in the hospital.

### 2.2. Data Collection

We conducted a thorough review of all electronic medical records to gather comprehensive data encompassing the demographic details, clinical presentations, laboratory findings, and comorbidities of each patient within the first 24 hours following their admission to the Intensive Care Unit (ICU). The collected laboratory parameters included a wide range of indicators such as white blood cell (WBC) count, neutrophil (NEU) count, lymphocyte (LYM) count, eosinophil (EOS) count, basophil (BAS) count, monocyte (MO) count, platelet (PLT) count, hematocrit (HCT) level, red blood cell count (RBC), neutrophil-to-lymphocyte ratio (NLR), platelet-to-neutrophil-to-lymphocyte ratio (PNLR), platelet-to-lymphocyte ratio (PLR), lymphocyte-to-monocyte ratio (LMR), C-reactive protein (CRP) level, D-dimer level, ferritin level, ferritin lactate (FL) index, alanine aminotransferase (ALT) level, aspartate aminotransferase (AST) level, total bilirubin (TB), albumin, globulin, albumin/globulin ratio (AGR) blood urea nitrogen (BUN), creatinine, calcium (Ca), chlorine (Cl), and sodium (Na).

In addition to laboratory results, we meticulously recorded each patient’s comorbidities, prescribed treatments, survival outcomes, and the duration of their hospital stay. The list of comorbidities specifically included hypertension (HT), diabetes mellitus (DM), chronic heart disease (CHD), chronic pulmonary disease (excluding asthma), and chronic kidney disease. To ensure the accuracy of COVID-19 diagnoses, RT-PCR test results were directly obtained from the Ministry of Health’s Public Health Management System.

### 2.3. Definition of Indexes

**Figure 1:**
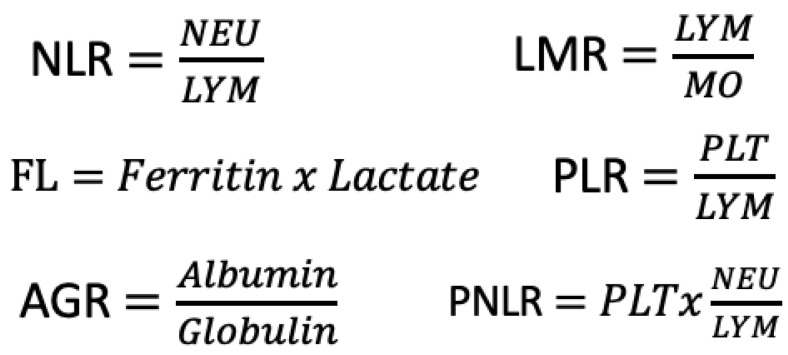
Definition of indexes.

NLR: The neutrophil-to-lymphocyte ratio was calculated by dividing the number of neutrophils by the number of lymphocytes. PLR: The platelet-to-lymphocyte ratio was calculated by dividing the number of platelets by the number of lymphocytes. PNLR: The platelet-to-neutrophil-to-lymphocyte ratio was obtained by multiplying the platelet count by the neutrophil count and then dividing by the lymphocyte count. LMR: The lymphocyte-to-monocyte ratio was determined by calculating the ratio of the lymphocyte count to the monocyte count. AGR: The albumin-to-globulin ratio was derived by comparing the level of albumin to the level of globulin. FL index: The ferritin lactate index was generated by multiplying the ferritin level by the lactate level (Figure 1).

### 2.4. Statistical Analysis

This study utilized Jamovi v2.4.1 for analysis, computing means, standard deviations, and frequencies for statistical examination. The Shapiro–Wilk test assessed the normality of data distribution. Continuous data between groups were compared using the independent sample *t*-test, while the Chi-Square and Fisher Exact tests evaluated associations between categorical variables. A binary logistic regression analysis developed a COVID-19 mortality model. For the parameters BUN, creatinine, albumin, globulin, D-dimer, Na, K, Ca, Cl, AST, ALT, TB, and AGR, data were missing for 16% of the patients (n = 33), and therefore, this missing information was omitted from the analysis in the model. Receiver Operating Characteristic (ROC) analysis calculated the variables’ sensitivity, specificity, cut-off values, and area under the curve (AUC). Youden’s index was used for the calculation of cut-off values. Statistical significance was set at *p* < 0.05.

## 3. Results

A total of 204 patients were included. Patients in Group 2 (deceased) had a notably higher mean age of 76 ± 11 years compared to those in Group 1 (survivors), whose mean age was 66 ± 15 years, indicating a statistically significant difference (*p* < 0.001). When examining gender distribution, we found no significant difference between the groups (*p* = 0.396), with males accounting for 49.2% (n = 64) of Group 1 and 55.4% (n = 41) of Group 2. Females comprised 50.8% (n = 66) of Group 1 and 44.6% (n = 33) of Group 2. The investigation into comorbidities revealed no statistically significant differences between the two groups (*p* > 0.05 for each comparison) (Table 1).

Procalcitonin was notably higher in Group 2 (median 0.95, Q1–Q3: 0.16–4.8) compared to Group 1 (median 0.14, Q1–Q3: 0.07–0.31), with a *p*-value of <0.001. D-dimer levels were significantly elevated in Group 2 (median 1355, Q1–Q3: 846–3355) in comparison to Group 1 (median 1040, Q1–Q3: 683–1492) (*p* = 0.005). Ferritin showed a significant increase in Group 2 (median 729, Q1–Q3: 355–1520) versus Group 1 (median 458, Q1–Q3: 254–875), with a *p*-value of 0.002. NLR was significantly higher in Group 2 (median 6.13, Q1–Q3: 3.5–12.8) compared to Group 1 (median 4.28, Q1–Q3: 2.6–7.5) (*p* < 0.001). PLR also showed a significant difference, with Group 2 (median 206, Q1–Q3: 133–336) having higher values than Group 1 (median 161, Q1–Q3: 126–242) (*p* = 0.006). PNLR exhibited a significant difference, with Group 2 showing higher levels (median 1272, Q1–Q3: 660–2792) compared to Group 1 (median 934, Q1–Q3: 502–2052) (*p* = 0.001). AGR was significantly lower in Group 2 (median 0.80, Q1–Q3: 0.7–0.9) than in Group 1 (median 0.94, Q1–Q3: 0.8–1.1) (*p* < 0.001). The FL index, an indicator of coagulation and inflammation, was significantly higher in Group 2 (median 1769, Q1–Q3: 830–3066) compared to Group 1 (median 756, Q1–Q3: 385–1593 (*p* < 0.001). The laboratory results are detailed in Table 2.

**Figure 2:**
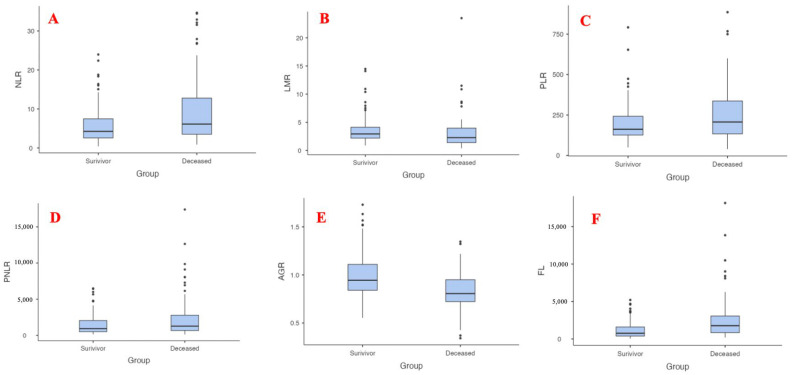
Average distributions of the indices according to groups. (**A**) Neutrophil-to-lymphocyte ratio (NLR); (**B**) Lymphocyte-to-monocyte ratio (LMR); (**C**) Platelet-to-lymphocyte ratio; (**D**) Platelet-to-neutrophil-to-lymphocyte ratio; (**E**) Albumin/globulin ratio; (**F**) Ferritin lymphocyte (FL) index.

The average distributions of the indices according to groups are given as a boxplot in Figure 2.

**Figure 3:**
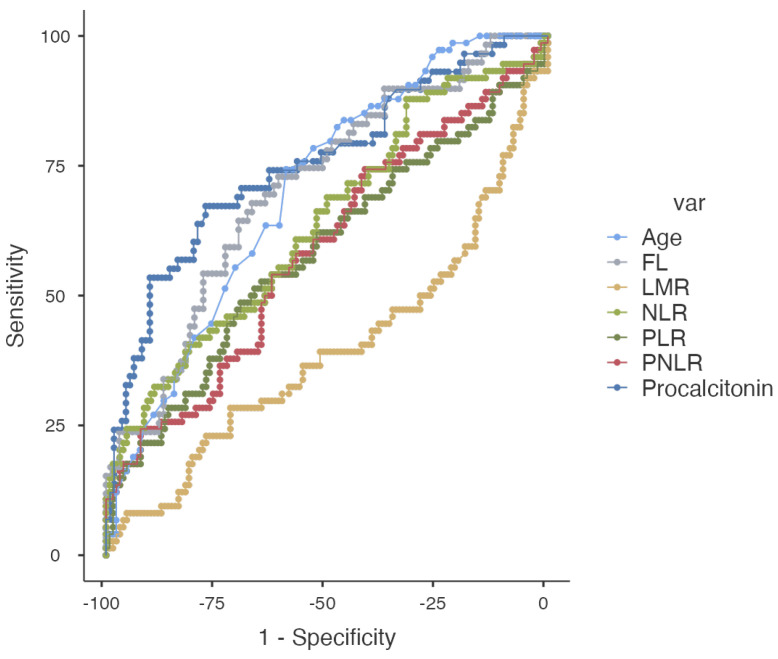
The ROC graph of mortality predictors.

With a cut-off value of 73 years, age demonstrated a sensitivity of 74% and a specificity of 60% for predicting mortality. The PPV and NPV were 50% and 80%, respectively. The AUC was 0.701. At a cut-off of 0.35 ng/mL, procalcitonin showed a sensitivity of 67% and a specificity of 77%, indicating a relatively balanced performance in identifying true positives and true negatives. The PPV and NPV were 60% and 82%, respectively, reflecting a solid performance in predicting mortality. The AUC was 0.752. With a cut-off value of 1228 mg/dL, FL had a sensitivity of 68% and a specificity of 65%. The PPV and NPV were 54% and 77%, respectively. The AUC was 0.707. With a cut-off value of 212, PLR had a sensitivity of 48% and a specificity of 69%. The PPV and NPV were 48% and 70%, respectively. The AUC was 0.582. With a cut-off value of 5.8 mg/dL, NLR had a sensitivity of 55% and a specificity of 63%. The PPV and NPV were 46% and 71%, respectively. The AUC was 0.640 (Table 3). The ROC analysis graph is given in Figure 3.

The statistical analysis evaluated the effects of age (>73), procalcitonin levels (>0.35), FL > 1228, PLR > 212, and NLR > 5.8 on mortality variables using logistic regression. Ages over 73 significantly increased event odds by 2.1 times (*p* = 0.05), procalcitonin levels above 0.35 nearly quintupled the odds (OR = 5.6, *p* < 0.001), high FL index levels more than tripled the odds (OR = 3.5, *p* = 0.003), a PLR > 212 significantly increased event odds by 3.5 (*p* = 0.030), and an NLR > 5.8 significantly increased event odds by 1.6 (*p* = 0.043) (Table 4).

**Figure 4:**
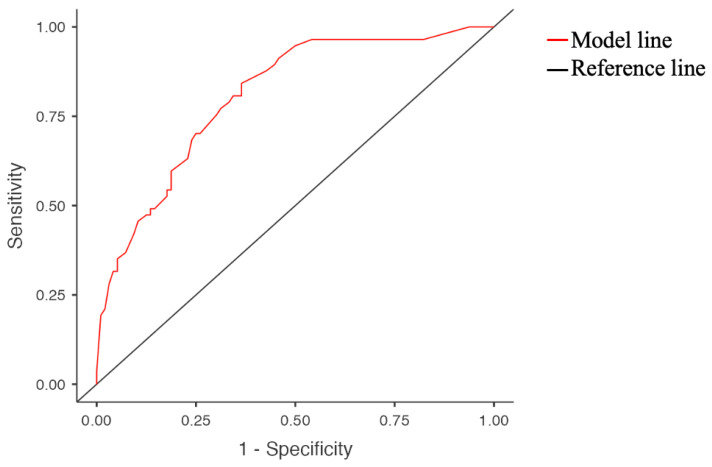
ROC graph of total mixed effects of predicted model.

Table 5 and Figure 4 demonstrate the total mixed effects of the predicted model.

## 4. Discussion

The COVID-19 pandemic has posed an unprecedented challenge to global health, revealing the need for a deeper understanding of its pathogenesis and determinants of disease severity. The virus’s ability to cause a spectrum of clinical manifestations, ranging from asymptomatic cases to severe, life-threatening illnesses, has underscored the importance of identifying factors that predict the course of the disease. Our study, focusing on an ICU patient cohort, provides further insights into the complex interaction between COVID-19 and patient-specific variables such as comorbidities and laboratory parameters.

Our research provides compelling evidence that certain demographic and laboratory markers can serve as significant predictors of mortality in COVID-19 patients. Advanced age and specific hematological changes, including elevated neutrophil counts, reduced lymphocyte levels, and increased NLR and PLR values, were associated with an increased risk of death. Notably, the FL index, as a new prognostic marker, demonstrated a substantial correlation with mortality outcomes, suggesting its potential utility in clinical assessments. The statistical analysis, using logistic regression, indicated that ages greater than 73, procalcitonin levels above 0.35, ferritin levels over 1228, PLR levels above 212, and NLR levels above 5.8 significantly increased the odds of mortality in COVID-19 patients. Specifically, patients older than 73 had more than double the risk of mortality, those with higher procalcitonin levels had nearly six times the risk, and an elevated FL index was associated with more than triple the mortality risk.

CRP is a protein synthesized by the liver and its levels rise in the presence of inflammation [26]. Typically, CRP concentrations are considerably higher in bacterial infections as compared to viral ones. Intriguingly, a notable elevation in CRP levels was observed among the COVID-19 patients in some studies, corroborating findings from other research [26,27,28]. However, our study did not find a significant difference in CRP levels between groups.

Numerous scholarly investigations have established that an elevated NLR at the time of hospital admission is a hallmark of severe COVID-19 cases, profoundly exceeding the levels found in patients experiencing mild to moderate disease presentations, and is concomitantly associated with an escalated risk of mortality [28,29]. Our research corroborates these observations, revealing a pronounced augmentation in the NLRs among the cohort of patients who ultimately succumbed to COVID-19 relative to those who recovered. The NLR and PLR have been recognized as an efficacious, standalone proxy for the assessment of systemic inflammatory activity. Neutrophils are integral to the innate immune defense, and their overactivation can precipitate multi-organ failure and mortality in severely compromised patients. On the other hand, lymphocytes are instrumental in regulating the inflammatory response. Consequently, an elevated NLR is indicative of a dysregulated immune response and could potentially serve as a prognostic biomarker for disease severity in infectious pathologies such as sepsis and bacteremia, reflecting the intensity of the host’s response to the pathogen [30].

Increased blood lactate concentration (hyperlactatemia) is common in critically ill patients. Arterial blood lactate levels are routinely measured in the ICU to estimate disease severity, predict morbidity and mortality, indicate specific treatments, and monitor the adequacy and timing of interventions [31]. In our study, the FL index was found to be higher in patients who died, and it was seen as a predictive factor of mortality compared to binary logistic regression analysis.

Serum ferritin, a widely recognized biomarker for assessing iron stores, also serves as a prognostic indicator of mortality in several critical conditions, including hemodialysis and sepsis [32]. The elevation of ferritin, which is produced by hepatocytes and macrophages, is linked to the activation of these macrophages [33]. In the context of COVID-19, an unchecked inflammatory response can inflict extensive tissue damage, particularly in elderly patients with chronic illnesses such as CHD, HT, or DM, thereby contributing to an increased mortality rate [33]. Furthermore, substantial elevations in serum ferritin have been observed in patients who have died from COVID-19 [34]. Liu et al. determined that the ferritin/lymphocyte ratio (FLR) holds prognostic significance in COVID-19 patients and is distinct from other inflammatory markers like C-reactive protein (CRP) and white cell count (WCC). FLR shows a high degree of sensitivity and negative predictive values for predicting adverse clinical outcomes in COVID-19, suggesting it could serve effectively as a “rule-out” test [35]. Consistent with these findings [34], our study found that the FL index was significantly elevated in patients who did not survive the infection compared to those who recovered. In patients with COVID-19, we observe an increase in both ferritin and lactate levels, and as the levels of these two markers rise, the severity of COVID-19 increases. The simultaneous elevation of both parameters in our study is significant. Based on these increases, we calculated the FL index. An FL index greater than 1228 was found to be associated with mortality. Our findings indicate that the FL index provides a more substantial increase and more predictive outcomes than the individual increases in ferritin and lactate. In this context, our study is the first of its kind in the literature.

Albumin and globulin are two important components of serum proteins and have been proven to be involved in systemic inflammation. Low serum albumin reflects malnutrition and liver and kidney dysfunction. İt has been shown to be an independent predictor of poor survival in critically ill patients. Reduced albumin at admission was an independent risk factor associated with no improvement at follow-up in COVID-19 patients [36,37]. On the other hand, increased globulin levels may reflect a chronic inflammatory response. Thus, the additive effect of both albumin and globulin will not only be a prognostic factor for possible complications of COVID-19 during the course of the disease but also an initial risk index for SARS-CoV-2-positive individuals [36,37]. In our study, the albumin level and AGR were significantly lower in patients who died, but unlike other studies, an increase in globulin level was a predictor of mortality.

In a study, it was found that CX3CL1, D-dimer, procalcitonin, and Interleukin-6 could effectively predict mortality in patients with severe COVID-19 [38]. Moreover, only the circulating levels of CX3CL1 and D-dimer were significantly correlated with the duration of illness. Zhao et al also observed high levels of D-dimer in patients who died due to COVID-19 [39]. Similarly, our research identified elevated D-dimer levels in patients who succumbed to COVID-19.

In their study, Nie et al. identified that LYM screening upon admission serves as a critical predictor for evaluating disease severity and clinical outcomes in COVID-19 patients, and they found a substantial correlation between lymphopenia and poor clinical outcomes [40]. Similarly, in our study, we observed that patients with lymphopenia experienced worse clinical courses. Thus, our research also demonstrates similarities with the existing literature in this regard.

PCT, a glycoprotein that serves as the precursor to calcitonin, lacks hormonal activity [41,42]. Typically, serum PCT levels are low or not detectable. Elevated PCT levels are indicative of bacterial infections, whereas they remain comparatively lower in viral infections, making PCT a useful marker for differentiating between bacterial and viral infections. The observation of higher PCT levels in severely ill COVID-19 patients suggests the possibility of coexisting bacterial infections. Liu et al. determined an optimal PCT cut-off value of 0.07 ng/mL, which falls within the normal range of 0–0.5 ng/mL [43]. In our study, at a cut-off of 0.35 ng/mL, procalcitonin showed a sensitivity of 67% and a specificity of 77%, indicating a relatively balanced performance in identifying true positives and true negatives. The PPV and NPV were 60% and 82%, respectively, reflecting a solid performance in predicting mortality. The AUC was 0.752.

A major strength of this study is its pioneering analysis of the FL index, revealing its potential as a novel prognostic marker for COVID-19 severity. The study’s limitations stem primarily from its retrospective design and the specific setting of a single COVID-19 Intensive Care Unit (ICU), which may restrict the generalizability of the findings to broader populations and different healthcare environments. Additionally, the reliance on medical records for data collection could introduce bias, as the completeness and accuracy of these records can vary. The study’s observational nature also means that causal relationships cannot be definitively established between the identified predictors and mortality outcomes. Furthermore, the exclusion of certain patient groups, such as those with negative RT-PCR results or those transferred to other facilities, may lead to selection bias. Another limitation of the study is that data for the parameters BUN, creatinine, albumin, globulin, D-dimer, Na, K, Ca, Cl, AST, ALT, TB, and AGR were missing for 16% of the patients (n = 33). Consequently, these gaps in the data were excluded from the model’s analysis. Finally, the study does not account for potential confounders, or the impact of therapeutic interventions received during the ICU stay, which could influence the outcomes. These limitations highlight the need for prospective, multi-center studies to validate the identified predictors and explore the mechanisms underlying the associations with mortality in COVID-19 patients.

## 5. Conclusions

Our study highlights significant predictors of mortality in COVID-19 ICU patients, including advanced age, elevated procalcitonin, FL index levels, the PLR, and the NLR. These findings contribute to the early identification and management of high-risk patients, underscoring the need for further research to validate these predictors and enhance patient care strategies.

## Figures and Tables

**Table 1:** Demographics.

Characteristics	Group 1 (n = 130)	Group 2 (n = 74)	*p*-Value
Age (year)	66 ± 15	76 ± 11	<0.001
Gender			0.396
Male	64 (49.2%)	41 (55.4%)	
Female	66 (50.8%)	33 (44.6%)	
Comorbidity			
HT	44 (38.8%)	28 (37.8%)	0.566
Diabetes	36 (27.7%)	13 (17.6%)	0.104
CHD	25 (19.2%)	16 (21.6%)	0.682
CPD	7 (5.4%)	4 (5.4%)	0.995
CKD	2 (1.5%)	1 (1.4%)	0.915

HT: Hypertension; CHD: Chronic heart disease; CPG: Chronic pulmonary disease; CKD: Chronic kidney disease.

**Table 2:** Comparison of laboratory and index results.

	Group 1 (n = 130)	Group 2 (n = 74)	
	Median (Q1–Q3)	Median (Q1–Q3)	*p*-Value
WBC	6.89 (5.2–10.2)	8.59 (5.7–12.3)	0.111
NEU	5.20 (3.6–8.2)	6.84 (4.1)	0.038
LYM	1.31 (0.9–1.6)	0.97 (0.7–1.4)	<0.001
EOS	0.01 (0–0.03)	0.01 (0–0.02)	0.201
BAS	0.02 (0.01–0.03)	0.02 (0.01–0.03)	0.505
MO	0.39 (0.28–0.6)	0.44 (0.3–0.6)	0.581
PLT	217 (169–269)	197 (156–245)	0.163
RBC	4.69 (4.4–5.1)	4.60 (4.1–5.2)	0.324
HGB	13.45 (12.5–14.8)	13.60 (12.1–15.2)	0.938
HCT	41.60 (38.2–44.8)	41.75 (37.5–46.9)	0.627
BUN *	19.10 (13.5–26.9)	27.05 (20.1–37.7)	<0.001
Creatinine *	1.00 (0.83–1.31)	1.28 (0.9–1.7)	<0.001
Albumin *	34.50 (31–38)	31 (28–35)	<0.001
Globulin *	35 (32–40)	38 (34.3–41.2)	0.001
Lactate	1.75 (1.4–2.3)	2.1 (1.6–3.3)	<0.001
CRP	69.8 (27.5–112.4)	70.2 (33.9–148.1)	0.220
Procalcitonin	0.14 (0.07–0.31)	0.95 (0.16–4.8)	<0.001
D-dimer *	1040 (683–1492)	1355 (846–3355)	0.005
Ferritin	458 (254–875)	729 (355–1520)	0.002
Na *	139 (135–143)	140 (136–144)	0.138
K *	4.2 (3.8–4.6)	4.3 (3.9–4.7)	0.712
Ca *	1.15 (1.1–1.2)	1.16 (1.1–1.2)	0.475
Cl *	102 (99–106)	103 (99–107)	0.304
AST *	39 (26.2–51)	37 (28.3–52.8)	0.864
ALT *	24 (16.2–34.8)	19.5 (14–31.8)	0.108
TB *	0.51 (0.4–0.8)	0.64 (0.5–1)	0.018
NLR	4.28 (2.6–7.5)	6.13 (3.5–12.8)	<0.001
PLR	161 (126–242)	206 (133–336)	0.006
LMR	2.94 (2.2–4.1)	2.28 (1.4–3.9)	0.013
PNLR	934 (502–2052)	1272 (660–2792)	0.001
AGR *	0.94 (0.8–1.1)	0.80 (0.7–0.9)	<0.001
FL	756 (385–1593)	1769 (830–3066)	<0.001

WBC: White blood cell count, NEU: Neutrophil count, LYM: Lymphocyte count, EOS: Eosinophil count, BAS: Basophil count, MO: Monocyte count, PLT: Platelet count, HGB: Hemoglobin, HCT: Hematocrit level, RBC: Red blood cell count, NLR: Neutrophil-to-lymphocyte ratio, PNLR: Platelet-to-neutrophil-to-lymphocyte ratio, PLR: Platelet-to-lymphocyte ratio, LMR: Lymphocyte-to-monocyte ratio, CRP: C-reactive protein level, ALT: Alanine aminotransferase level, AST: Aspartate aminotransferase level, TB: Total bilirubin, AGR: Albumin/globulin ratio, BUN: Blood urea nitrogen, Ca: Calcium, Cl: Chlorine, Na: Sodium, FL: Ferritin lactate index. Note: * Data were missing for 16% of patients (n = 33); thus, these data were excluded from the model analysis.

**Table 3:** Mortality predictors and their ROC analysis results.

	Cut-Off	Sensitivity (%)	Specificity (%)	PPV (%)	NPV (%)	AUC
Age	73	74%	60%	50%	80%	0.701
Procalcitonin	0.35	67%	77%	60%	82%	0.752
FL	1228	68%	65%	54%	77%	0.707
NLR	5.8	55%	63%	46%	71%	0.640
PLR	212	48%	69%	48%	70%	0.582
PNLR	1195	52%	61%	44%	69%	0.579
LMR	4.42	18%	79%	35%	62%	0.395

FL: Ferritin lactate index, NLR: Neutrophil-to-lymphocyte ratio, PLR: Platelet-to-lymphocyte ratio, PNLR: Platelet-to-neutrophil-to-lymphocyte ratio, LMR: Lymphocyte-to-monocyte ratio.

**Table 4:** Regression analysis results for mortality.

						95% Confidence Interval
Predictor	Estimate	SE	*p*	Odds Ratio	Lower	Upper
Intercept	−2.4912	0.595	<0.001	0.08	0.03	0.26
Age > 73	0.7401	0.401	0.05	2.1	1.1	4.6
Procalcitonin > 0.35	1.7195	0.436	<0.001	5.6	2.37	13.1
FL > 1228	1.2601	0.423	0.003	3.5	1.5	8.1
NLR > 5.8	0.586	0.303	0.043	1.6	1.06	2.5
PLR > 212	1.2556	0.540	0.020	3.5	1.2	10.1
PNLR > 1195	−0.1949	0.743	0.793	0.8	0.12	3.5
LMR < 4.42	−0.0504	0.558	0.928	0.9	0.32	2.8

FL: Ferritin lactate index, NLR: Neutrophil-to-lymphocyte ratio, PLR: Platelet-to-lymphocyte ratio, PNLR: Platelet-to-neutrophil-to-lymphocyte ratio, LMR: Lymphocyte-to-monocyte ratio.

**Table 5:** Total mixed effects of predicted model.

Accuracy	Specificity	Sensitivity
0.712	0.823	0.526

Note. The cut-off value is set to 0.5.

## Data Availability

The datasets generated during and/or analyzed during the current study are available from the corresponding author upon reasonable request.
